# The role of the innate immune system in allergic contact dermatitis* 

**DOI:** 10.5414/ALX01274E

**Published:** 2017-08-04

**Authors:** S.F. Martin

**Affiliations:** Allergy Research Group, Department of Dermatology, University Medical Center, Freiburg, Germany

**Keywords:** skin, contact allergen, innate immune system, inflammation, dendritic cell, Toll-like receptor, inflammasome, oxidative stress

## Abstract

Abstract. Allergic contact dermatitis is a Tcell mediated inflammatory skin disease that is caused by low molecular weight chemicals and metal ions. These contact allergens induce skin inflammation, an essential element of the sensitization process. Our understanding of the molecular mechanisms that underlie chemical-induced inflammation has improved significantly over the last years. The emerging picture shows that contact allergens activate known innate immune and stress responses that play a role in immune responses to infections. Contact allergens use innate immune receptors such as the Toll-like receptors TLR2 and TLR4 and the NOD-like receptor NLRP3 as part of the inflammasome as well as the induction of oxidative stress to induce skin inflammation. The detailed identification of the relevant signaling pathways and the mechanisms of their activation by contact allergens will most likely lead to more targeted therapeutic approaches by interference with these pathways. Moreover, this will help to refine existing, and to develop new in vitro assays for the identification of contact allergens, an important step to replace animal testing e.g. for ingredients of cosmetics which has been prohibited now by EU legislation.

German version published in Allergologie, Vol. 33, No. 2/2010, pp. 66-70

*According to a lecture on the occasion of the 4th German Allergy Congress, Berlin, September 3 – 6, 2009.

**Abbreviations:** DC: dendritic cell; TLR: Toll-like receptor; NLR: NOD-like receptor; PTM: post-translational modification.

## Mechanisms of allergic contact dermatitis 

Contact allergens are low molecular weight chemicals and metal ions. Skin contact can cause a hypersensitivity reaction, i.e. allergic contact dermatitis, in some human subjects. Approximately 4,000 of these contact allergens are known and they can be found virtually everywhere in our environment in the form of natural, e.g. plant-derived, or synthetic substances [1, 2]. The first contact with a contact allergen leads to sensitization, further contacts cause induction of an inflammatory eczematous reaction in the skin. The ability of contact allergens to cause skin inflammation is of central importance for sensitization [3, 4]. In this process the binding of contact allergens to as yet largely unknown target proteins is essential. This is also necessary in order to make the contact allergens recognizable for the T and B cells of the adaptive immune system, as these haptens in their unbound form are not immunogenic. 

It is the inflammatory response in the skin that enables epidermal Langerhans cells and dermal dendritic cells (DC) to mature and emigrate. These cells carry the contact allergens from the skin into the draining lymph nodes where they activate naïve T cells. With their T cell receptor these recognize the contact allergens with high specificity on the dendritic cells in the context of MHC molecules [2, 16]. Then the T cells divide, become effector and memory T cells and are recruited to the inflamed skin after there had been skin contact with the allergen again. The targeted migration into the skin becomes possible by the fact that dendritic cells from the skin upregulate a skin-specific combination of so-called homing receptors on the allergen-specific T cells in the lymph node . These are the adhesion molecule CLA (Cutaneous Lymphocyte Antigen) and the chemokine receptors CCR4 and CCR10. [5, 6, 7]. Both the tissue micro environment of the skin and of the draining lymph nodes, are important for this so-called imprinting of homing receptors [8, 9, 10, 11]. In the skin the cytotoxic activity and cytokine production of the immigrated effector T cells against contact allergen-presenting skin cells lead to the well-known clinical picture of allergic contact dermatitis. 

## Inflammation of the skin caused by chemicals and metal ions 

**Figure 1.**
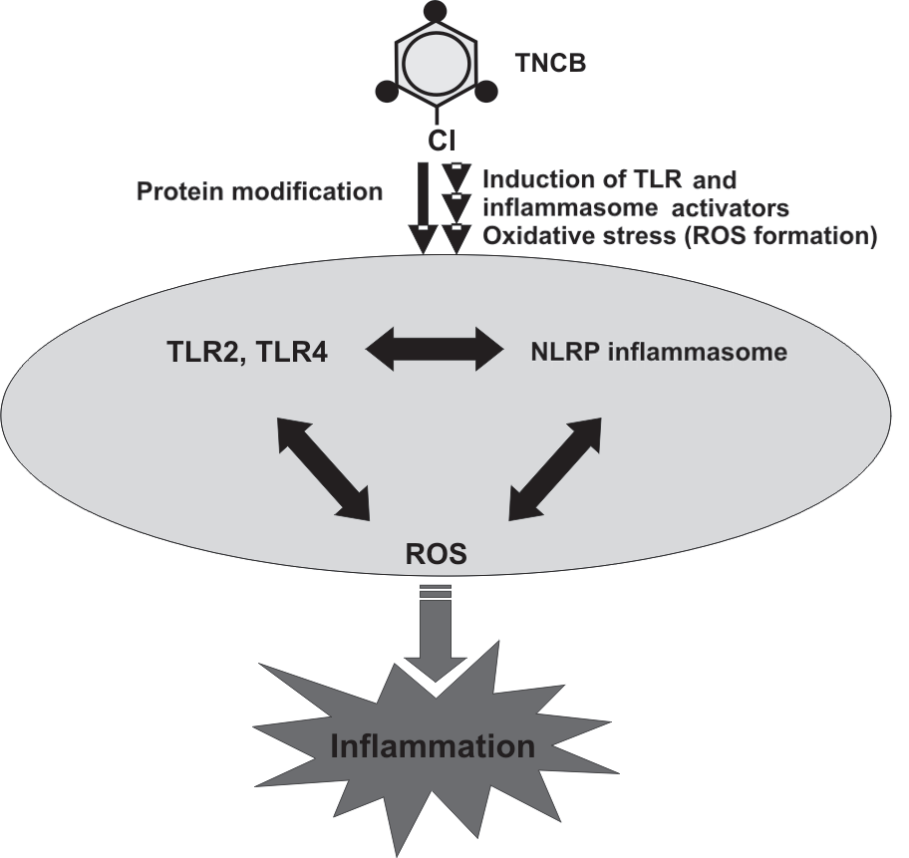
Activation of innate immune and stress responses by contact allergens. Contact allergens like TNCB induce the formation of activators for TLR2 and TLR4 as well as for the NLRP3 inflammasome by modification of so-far unknown target proteins. Additionally, oxidative stress responses cause the formation of reactive oxygen species (ROS). These processes result in an inflammation of the skin that is critical for successful sensitization and the activation of T cell response against contact allergens.

In the past years the molecular mechanisms of the triggering of inflammation by contact allergens have been better understood [3, 4] Here, a fascinating analogy between contact allergens and infectious agents is becoming evident. They activate similar signaling pathways but do so via different mechanisms (Figure 1). 

For infectious agents it is known that components of their cell walls and nucleic acids, collectively designated pathogen associated patterns (PAMPs), bind to pattern recognition receptors (PRR) of the innate immune system thereby activating signaling pathways that lead to inflammation. These receptors belong to protein families. The best known are the Toll-like receptors (TLR) [12, 13] and the NOD-like receptors (NLR) [14, 15]. TLR are localized in the plasma membrane or in the membranes of endosomal compartments. TLR4 is the best-known TLR and recognizes bacterial lipopolysaccharide (LPS) that plays an important role in cases of septic shock. NLRs are localized in the cytosol. NOD2, a well-known NLR, recognizes bacterial peptido glycans. Another NLR, NLRP3, plays an important role in inflammatory processes [15]. It forms a cytosolic complex with the adapter protein ASC, the so-called NLRP3 inflammasome, which is responsible for activation of caspase-1. Amlong other activities, this protease generates the mature forms of the cytokines IL-1β and IL-18 which are produced as immature pro-forms after activation of e.g. TLR4 and which are important inflammatory mediators. The NLRP3 inflamma some, is activated by bacterial toxins and other danger signals [15]. 

Based on th knowledge regarding mechanism of inflammtion triggered by infectious agents, we and others addressed the question whether contact allergen induced inflammation also uses these pathways. Studies in the mouse model of contact dermatitis, the contact hypersensitivity (CHS) model [16], showed that the simultaneous absence of TLR2 and TLR4 leads to resistance against potent contact allergens like TNCB and oxazolone [17]. The selective absence of these TLR on dendritic cells was sufficient to completely inhibit sensitization. Interestingly, evidence for the activation of TLR2 and TLR4 by non-microbial, i.e. endogenous ligands was found. Accordingly, degradation products of the extracellular matrix component hyaluronic acid (HA) were identified as putative ligands for these TLR in CHS [4, 17, 18, 19]. The use of receptors of the innate immune system by endogenous ligands seems to be a common principle. This could be the basis for some sterile inflammatory reactions . Endogenous activators have also been described for the NLRP3 inflammasome [15]. Among them are: extracellular ATP which is released by stressed and damaged cells; uric acid crystals which, in gout, cause inflammation by inflammasome activation; exogenous substances like asbestos, silica crystals or aluminum hydroxide, which is used as an adjuvant in some vaccines. The CHS model showed a significant reduction in contact allergy when NLRP3, ASC or Caspase-1 were absent [20, 21, 22, 23]. By using the TLR and NLR systems contact allergens obviously use pathways that are normally responsible for anti-infectious responses. But what are the molecular mechanisms of activation? For TLR2 and TLR4 as well as for NLRP3 there seem to be endogenous activators the formation and release of which is obviously caused by contact allergens [4, 17]. It might, however, also be possible that some contact allergens activate signaling pathways by binding directly to these receptors [4, 24]. 

Here we propose two concepts for activation of the innate immune system by contact allergens:

indirect activation by formation/release of endogenous ligands for pattern recognition receptors and
direct activation by chemical modification of these receptors or of other components of the respective signaling path ways.

## Hapten modification and possible analogy to post-translational modifications 

For the direct activation of signaling pathways we postulate that the proven binding of contact allergens to amino acids like cysteine and lysine in proteins can cause a modification of protein function and/or localization within the cell, just as classical post- trans lational modifications (PTM) do [4, 24]. Examples for this are phosphorylation and glycosylation. Comparing these hapten modifications of proteins with PTM might be attractive but still remains to be proven in further investigations. 

An example in support of our PTM hypothesis is the activation of the so-called antioxidant phase 2 response of cells. The covalent modification of the cytosolic sensor protein Keap1 is carried out by cysteine-binding chemicals [25, 26]. Keap1 associates with the transcription factor Nrf2 and inactivates it by mediating its ubiquitination and degradation by the proteasome. When a contact allergen binds to Keap1, Nrf2 is released. After translocation into the nucleus, it can activate genes that carry a Nrf2 binding site in their promoter [25]. Among these are genes for well-known antioxidative proteins as for example glutathione synthetase, catalase, super oxide dismutase, hemoxygenase and nicotinamide quinone oxidoreductase [27, 28, 29]. 

This response can also be induced by oxidative stress that leads to the oxidation of the thiol groups of Keap1 and thus to the Nrf2 translocation and gene activation [31]. In case of such stress-induced responses reactive oxygen species (ROS) are formed that play an important role in inflammatory processes and are produced in cells when phagocytosis of bacteria takes place [32, 33]. Interestingly, oxidative stress is not only caused by infectious agents but also by contact allergens [34, 35, 36]. 

This casts a new light on the inflammation of the skin in cases of contact dermatitis. Oxidative stress and the activation of TLR2 and TLR4 as well as of the NLRP3 inflam ma some are innate defense mechanisms that are triggered by contact allergens [4]. In this context it is important to note that these do not function independently but influence each other [37, 38]. This so-called cross-talk opens up the chance to therapeutically influence several signaling pathways by interfering with only one of them. Findings from the CHS model show that this might be possible. In the CHS model the loss of TLR2 and TLR4 leads to resistance against contact dermatitis, i.e. either the oxidative stress and the in flammasome pathways alone are not sufficient to permit contact dermatitis or these pathways are not efficiently activated without these TLR [17]. Similar results are seen for mice with inflammasome defects [20, 21, 22]. On the other hand it might, however, also be possible that the absence of endogenous activators of these mechanisms can be compensated by microbial ligands or weak inflammatory responses may be increased above a critical threshold by coincident infection. The latter may result in contact dermatitis to weak allergens. (This implies that infectious agents can be important triggering or co-factors for contact dermatitis. 

## Summary 

As more and more molecular mechanisms of inflammation caused by contact allergens are being understood, it is becoming obvious that these small molecules indirectly or directly activate the mechanisms of the innate immune system. In this context pattern recognition receptors like TLR2, TLR4 and NLRP3 but also oxidative stress and antioxidant responses that are also responsible for infection defense play an important role. The recognition of obvious analogies between infectious agents and contact allergens can be used in order to block the before-mentioned signaling pathways using specific therapeutic approaches and thereby avoid sensitization as shown in the CHS model (Esser P.R. et al., manuscript in preparation) and possibly also the induction of contact dermatitis. Furthermore, the detailed understanding of the molecular mechanisms can be used in order to improve in vitro testing and to develop new tests for the identification of contact allergens in the risk assessment for, e.g., ingredients of cosmetics and household products. This is urgently needed in order to avoid animal testing which is now prohibited for thecosmetics industry due to recent EU legislation (for further information see www.sens-it-iv.eu) [39].
